# Evaluation of Epithelial Integrity with Various Transepithelial Corneal Cross-Linking Protocols for Treatment of Keratoconus

**DOI:** 10.1155/2014/614380

**Published:** 2014-08-12

**Authors:** Suphi Taneri, Saskia Oehler, Grace Lytle, H. Burkhard Dick

**Affiliations:** ^1^Center for Refractive Surgery, Eye Department at St. Franziskus Hospital, Muenster, Hohenzollernring 70, 48145 Muenster, Germany; ^2^Eye Clinic, Ruhr University, Bochum, Germany; ^3^Avedro, Waltham, MA 02451, USA

## Abstract

*Purpose*. Corneal collagen cross-linking (CXL) has been demonstrated to stiffen cornea and halt progression of ectasia. The original protocol requires debridement of central corneal epithelium to facilitate diffusion of a riboflavin solution to stroma. Recently, transepithelial CXL has been proposed to reduce risk of complications associated with epithelial removal. Aim of the study is to evaluate the impact of various transepithelial riboflavin delivery protocols on corneal epithelium in regard to pain and epithelial integrity in the early postoperative period.* Methods*. One hundred and sixty six eyes of 104 subjects affected by progressive keratoconus underwent transepithelial CXL using 6 different riboflavin application protocols. Postoperatively, epithelial integrity was evaluated at slit lamp and patients were queried regarding their ocular pain level.* Results*. One eye had a corneal infection associated with an epithelial defect. No other adverse event including endothelial decompensation or endothelial damage was observed, except for epithelial damages. Incidence of epithelial defects varied from 0 to 63%. Incidence of reported pain varied from 0 to 83%.* Conclusion*. Different transepithelial cross-linking protocols have varying impacts on epithelial integrity. At present, it seems impossible to have sufficient riboflavin penetration without any epithelial disruption. A compromise between efficacy and epithelial integrity has to be found.

## 1. Introduction

Corneal collagen cross-linking (CXL) is the only conservative therapy for keratoconus that has been demonstrated to stiffen the cornea and halt the progression of the ectasia. CXL results in an increase in tensile strength of the cornea as a result of an interaction between riboflavin photosensitizer and ultraviolet light, which results in an increase in covalent bonding within or between collagen fibers that make up the anterior stromal lamellae [1]. The conventional protocol described by Wollensak et al. requires debridement of the central 9 mm of the corneal epithelium to facilitate diffusion of a solution containing 0.1% riboflavin with 20% dextran T500 to the corneal stroma [2].

Recently, transepithelial or “epithelium-on” CXL with modified technique has been proposed to reduce the risk of complications associated with epithelial removal [3, 4]. Provided that sufficient effect is obtained, transepithelial CXL is highly desirable from both the patient's and the ophthalmologist's perspective because ideally this approach avoids the pain, risk of infection, transient visual impairment, and all other consequences and potential complications of epithelial debridement [5].

A number of modified riboflavin formulations have been introduced to facilitate diffusion through the corneal epithelium. To our knowledge, to date, there has been no comparison of transepithelial formulations to evaluate whether these goals of transepithelial CXL are met. The purpose of this short-term study is to evaluate and compare the impact of various transepithelial riboflavin delivery protocols on the corneal epithelium in regard to pain and epithelial integrity in the early postoperative period.

## 2. Patients and Methods

One hundred and sixty-six eyes of 104 subjects affected by progressive keratoconus underwent transepithelial CXL between 05/2011 and 12/2013 at the Center for Refractive Surgery, St. Francis Hospital, Münster, Germany. Inclusion criteria included keratoconus I–III according to the Amsler-Krumeich classification with documented progression in the previous 12 months, defined as an increase in maximum keratometry (K Max) or subjective cylinder of 1.00 diopter (D) or more or subjective deterioration of visual acuity. Exclusion criteria included endothelial decompensation, central corneal opacities, history of herpetic keratitis, active corneal infection, aphakia, concomitant ocular or systemic autoimmune disease, pregnancy, and breastfeeding. Informed consent was obtained from all patients.

### 2.1. Patient Examinations

All eyes were evaluated by slit lamp examination to assess the presence or absence of any epithelial defects on each postoperative day until the eye was quiet and the epithelium was unremarkable. Visibly loose epithelium was considered as defective. On the first postoperative day, all patients were queried if they had experienced ocular pain of any level since transepithelial CXL. At every following visit the patients were again asked if they had experienced any ocular pain since the last visit. Optical coherence tomography (OCT) was used to qualitatively assess riboflavin diffusion postoperatively in some patients.

### 2.2. Surgical Procedure

Riboflavin application procedure was determined by a stepwise optimization protocol using one of 6 treatment regimens. In all cases, riboflavin application and subsequent UVA irradiation were performed according to manufacturer recommendations for the use of the riboflavin formulation and recommended parameters for UVA irradiation. The riboflavin formulations used are presented in Table 1, with the corresponding UVA delivery device used for the study treatments.

### 2.3. Surgical Technique and Procedure

In all treatments, the subject was placed in a supine position. Preservative free anesthetic eye drops were administered preoperatively and a lid speculum was applied. The corneal epithelium was left intact, and riboflavin application and UVA treatment were performed according to one of six regimens described below and summarized in Table 2.

Postoperative care included the use of a soft bandage contact lens in all of the eyes in Groups 4–6. No bandage contact lens was used in Group 1 and no bandage contact lens was used in the first 5 of eyes of Groups 2 and 3, respectively. The use of BSCL was introduced after observing epithelial defects in the first 5 eyes of Groups 2 and 3 in order to minimize stress on the epithelium by lid movements.

In Group 1, Ricrolin TE (Sooft, Italy) was applied at a rate of 1 drop every 2 minutes for approximately 30 minutes. Riboflavin was not rinsed from the cornea, and 3 mW/cm^2^ of irradiance was applied to the cornea for 30 minutes, for a total energy dose of 5.4 J/cm^2^. During illumination the cornea was kept moist by further application of Ricrolin TE at a rate of 1 drop every 2 minutes.

In Group 2, Medio-Cross TE (Peschke Meditrade GmbH, Germany) was applied at a rate of 1 drop every 2 minutes for approximately 30 minutes. Riboflavin was not rinsed from the cornea, and 3 mW/cm^2^ of irradiance was applied to the cornea for 30 minutes, for a total energy dose of 5.4 J/cm^2^. During illumination the cornea was kept moist by further application of Medio-Cross TE at a rate of 1 drop every 2 minutes.

In Group 3, ParaCel (Avedro Inc., USA) was applied at a rate of 1 drop every 60 seconds for approximately 15 minutes. Riboflavin was rinsed from the cornea using BSS, and 45 mW/cm^2^ of irradiance was applied to the cornea for 2 minutes and 40 seconds, for a total energy dose of 7.2 J/cm^2^. No further ParaCel was applied during illumination.

In Group 4, Ricrolin+ (Sooft, Italy) was administered after applying preservative-free anesthetic eye drops 10 minutes, 5 minutes, and immediately before, while only one application of anesthetic eye drops was used in all other groups as recommended by the respective manufacturers. An iontophoresis technique was utilized with a constant current and two electrodes. A circular reservoir with a surrounding annular suction ring was affixed to the cornea during the procedure. A stainless steel grid inside this reservoir served as the cathode at a minimal distance from the cornea, and an anode was affixed to the subjects' forehead. The reservoir was filled with Ricrolin+ solution. The generator was used to apply a constant current of 1 mA for a period of 5 min. After the 5-minute impregnation period, 10 mW/cm^2^ of irradiance was applied to the cornea for 9 minutes for a total energy dose of 5.4 J/cm^2^.

In Group 5, a two-stage application procedure for ParaCel and VibeX Xtra (Avedro Inc., USA) was used. ParaCel was applied at a rate of 1 drop every 90 seconds for 3 minutes. The cornea was then rinsed with VibeX Xtra completely coating the cornea. Additional VibeX Xtra was applied at a rate of 1 drop every 60 seconds for 7 minutes. A total riboflavin soak time of 10 minutes was achieved. Forty five mW/cm^2^ of irradiance was continuously applied to the cornea for 2 minutes and 40 seconds, for a total energy dose of 7.2 J/cm^2^.

In Group 6, the same two-stage application procedure for ParaCel and VibeX Xtra was used as in Group 5. However, the irradiance was applied in a pulsed mode in which the UV light was alternately turned on for one second and turned off for one second. The total energy dose was 7.2 J/cm^2^.

## 3. Results

One hundred sixty-six eyes were treated with transepithelial CXL according to 6 treatment regimens, with 110 eyes in Group 1, 8 eyes in Group 2, 12 eyes in Group 3, 10 eyes in Group 4, 13 eyes in Group 5, and 13 eyes in Group 6. Minimum corneal thickness was 335 *μ*m in Group 1, 396 *μ*m in Group 2, 367 *μ*m in Group 3, 442 *μ*m in Group 4, 377 *μ*m in Group 5, and 460 *μ*m in Group 6, respectively.

There was no serious complication except for one eye in treatment protocol 2 that had a corneal infection associated with an epithelial defect.

**Figure 1:**
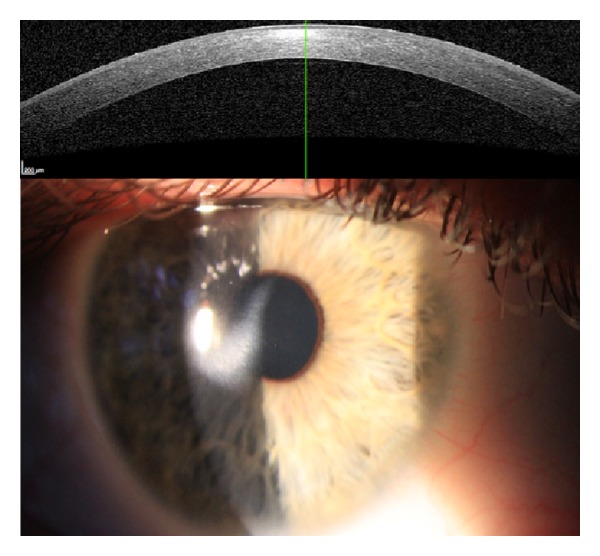
Paracentral subepithelial opacification after infection following Medio-Cross TE CXL.

Visual acuity was decreased to hand motion in the acute phase. After 18 months, central visual acuity was fully restored; however, a paracentral subepithelial opacification was still visible (Figure 1).

**Figure 2:**
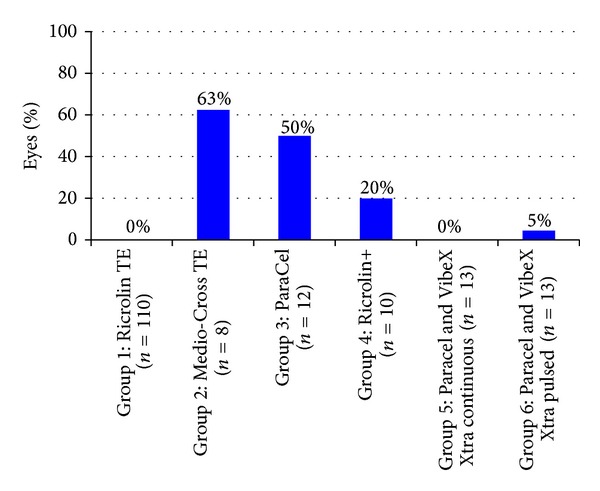
Percentage of eyes presenting with epithelial defect following transepithelial CXL.

No other adverse event including endothelial decompensation or endothelial damage was observed in any eye, except for epithelial damages. The incidence of postoperative epithelial defects according to treatment protocol is presented in Figure 2.

**Figure 3:**
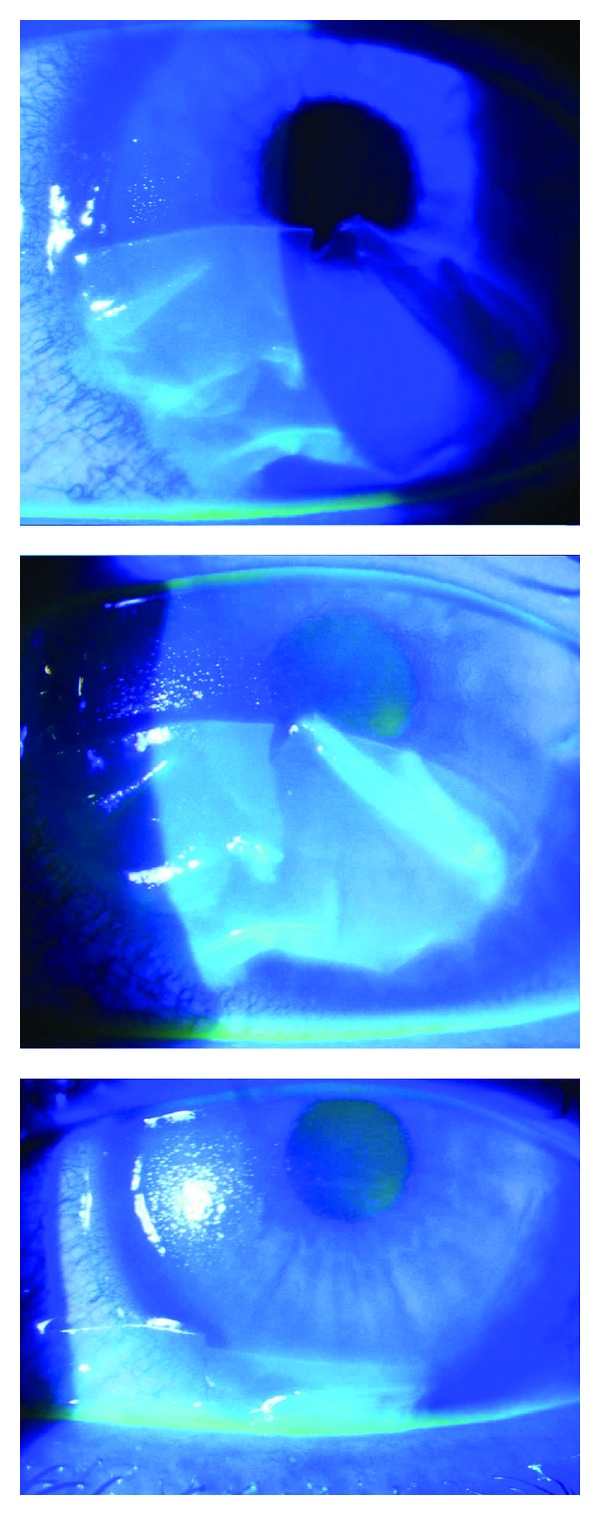
Epithelial sloughing after bandage contact lens removal, one day post-op transepithelial CXL with Medio-Cross TE.

Postoperative epithelial defects were most commonly observed on the first postoperative day. Often the complete illuminated epithelium was affected leading to a detachment as an intact sheet similar to a LASEK flap (Figure 3).

In some eyes, the epithelium was closed during the follow-up period. However, parts of it were loose and mobile over the corneal stroma leading to pain perception.

**Figure 4:**
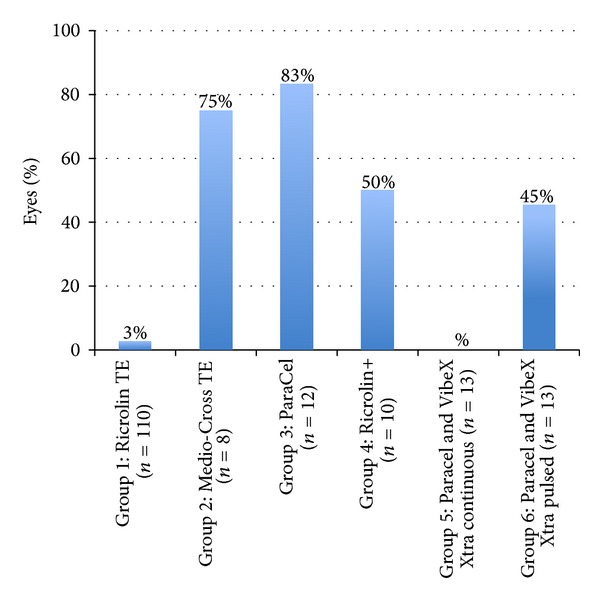
Percentage of eyes with postoperative pain following transepithelial CXL.

The incidence of reported postoperative pain is shown in Figure 4. In all groups, reported pain was the greatest in the 24 hours following the procedure, resolved by complete epithelial healing after 1–4 days.

OCT revealed limited or superficial hyperreflectivity in eyes treated according to the protocol for Group 1. OCT evaluation was comparable between the remaining groups, with deeper reaching hyperreflectivity observed in the corneal stroma in the postoperative period in Groups 2–6.

## 4. Discussion

Standard riboflavin formulations containing 0.1% riboflavin and 20% dextran show minimal penetration through intact or partially disrupted epithelium [6, 7]. The optimal approach for transepithelial CXL must minimize the impact on the corneal epithelium while permitting a sufficient amount of riboflavin to diffuse into the stromal tissue where cross-linking occurs. Epithelial disruption without full debridement leaves the cornea vulnerable to early postoperative infection and delays the return to gas permeable contact lens wear and visual recovery.

The results of this study reveal variability in postoperative recovery following transepithelial CXL with different treatment regimens. The use of Ricrolin TE resulted in the least disruption of the corneal epithelium, with no epithelial defects reported in any case and minimal postoperative discomfort. However, some epithelial disruption is necessary to allow diffusion of riboflavin to the corneal stroma. Reports assessing the diffusion of Ricrolin TE revealed a shallow penetration of the riboflavin which may be insufficient for cross-linking [5, 8, 9]. This finding prompted the exploration of further treatment protocols.

Qualitative evaluation of the depth of the riboflavin penetration with OCT revealed deeper penetration to the stroma following the remaining protocols in this study. However, variability was observed in the frequency of epithelial defects. Eyes treated with Ricrolin+ and Iontophoresis showed epithelial defects in 20% of eyes and pain in 50% of eyes. Based on our observation of eyes with apparently loose epithelium that leads to pain perception in the absence of an epithelial defect, we hypothesize that eyes experienced pain more often than they had epithelial defects because of subtle epithelial disruptions which were not detectable at slit lamp exam. Fifty percent of eyes in the ParaCel (alone) group and greater than 50% of eyes in the Medio-Cross TE group presented with epithelial defects in the first postoperative day.

Both the ParaCel and Medio-Cross TE formulations contain benzalkonium chloride, which acts as an epithelial permeability enhancer. The disruptive effects of BAC are both duration and concentration dependent [10], and therefore it is logical that reduction of the duration of exposure to BAC might reduce the incidence of epithelial defects. This was the rationale for the development of the two-stage riboflavin application method employing sequential application of 0.25% riboflavin with BAC (ParaCel) and 0.25% riboflavin without BAC (VibeX Xtra). According to a theoretical model proposed by Avedro, Inc., the initial soak with the riboflavin and BAC solution is sufficient to open the epithelial junctions and to provide the initial dose of riboflavin. Once the junctions have been sufficiently loosened, further exposure to BAC is not thought to provide any additional benefit, and it is flushed away. The remainder of the presoak time is completed using a BAC-free, dextran-free riboflavin solution [11].

The two-stage application appeared to be a near optimal protocol with respect to epithelial integrity, resulting in zero incidences of postoperative epithelial defects in Group 5 and a reduction in the percentage of eyes experiencing postoperative pain (0%) as compared to the use of ParaCel alone (83%). However, when pulsed, illumination was introduced to the treatment protocol of Group 5; that is, in Group 6, greater pain perception was observed. We may speculate that the prolonged treatment time may lead to desiccation of the ocular surface adding to the epithelial trauma.

While OCT evaluation of the depth of riboflavin penetration provides evidence of the efficacy of the two-stage application protocols, a clinical means of quantifying the concentration of riboflavin in the stroma as a function of depth would have added to this study. To our knowledge, no such technology currently exists. Therefore, longer term follow-up is necessary to evaluate the relative efficacy of these cross-linking protocols in regard to stabilization of the progression of keratoconus.

In conclusion, the findings of this study suggest that different transepithelial cross-linking protocols have varying impacts on epithelial integrity. At present, it seems impossible to have sufficient riboflavin penetration without any epithelial disruption. A compromise between efficacy and epithelial integrity has to be found. In children, it may be desirable to minimize discomfort and accept a less than maximum efficacy as the procedure may be repeated later on. In contrast, in very thin corneas, it may be an option to use an “aggressive” protocol to maximize efficacy even if the epithelium sloughs off postoperatively in order to have the epithelium as a protective spacer to the endothelium. Longer term outcomes of these various treatment protocols will follow and will provide insight into the selection of an appropriate treatment protocol for each of these patient scenarios.

## Figures and Tables

**Table 1:** Overview of the different riboflavin formulations, formulation compositions, and the UVA light source, and if iontophoresis was used in this study.

Riboflavin formulation	Formulation composition	UVA delivery device	Iontophoresis
Ricrolin TE (Sooft, Italy)	0.1% riboflavin-5-phosphate, 15% dextran T500, sodium edetate, trometamol, and NaCl	UV-X 1000, IROC Innocross, Switzerland	N/A

Medio-Cross TE (Peschke Meditrade GmbH, Germany)	0.25% riboflavin-5-phosphate hydroxypropyl methylcellulose, benzalkonium chloride, NaCl	UV-X 1000, IROC Innocross, Switzerland	N/A

ParaCel (Avedro Inc., USA)	0.25% riboflavin-5-phosphate, hydroxypropyl methylcellulose, sodium edetate, trometamol, benzalkonium chloride, NaCL	KXL, Avedro Inc., USA	N/A

Ricrolin+ (Sooft, Italy)	0.1% riboflavin-5-phosphate, sodium edetate, trometamol, sodium dihydrogen phosphate dihydrate, and sodium phosphate dibasic dehydrate	KXL, Avedro, Inc., USA	I-ON CXL generator (Sooft, Italy)

VibeX Xtra (Avedro Inc., USA)	0.25% riboflavin-5-phosphate and NaCl	KXL, Avedro, Inc., USA	N/A

**Table 2:** Overview of the 6 different treatment protocols used in this study.

Group	Riboflavin formulation	Soak time (minutes)	UVA irradiance (mW/cm^2^)	UVA time	Total energy (J/cm^2^)
1	Ricrolin TE	30	3	30 minutes	5.4
2	Medio-Cross TE	30	3	30 minutes	5.4
3	ParaCel	15	45	2 minutes 40 seconds	7.2
4	Ricrolin+ (with Iontophoresis)	5	10	9 minutes	5.4
5	ParaCel and VibeX Xtra (2 stage application)	3 + 7	45	2 minutes 40 seconds, continuous irradiation	7.2
6	ParaCel and VibeX Xtra (2 stage application)	3 + 7	45	5 minutes 20 seconds, pulsed irradiation (1 s on, 1 s off)	7.2
